# Comparative Study on Biological Characteristics and Functions of Three Porcine-Derived Lactic Acid Bacteria

**DOI:** 10.3390/ani16111732

**Published:** 2026-06-04

**Authors:** Miao Yu, Yaojun Li, Bing Yu, Daiwen Chen

**Affiliations:** 1 Animal Nutrition Institute, Sichuan Agricultural University, Chengdu 611130, China; yumiao314@foxmail.com (M.Y.); ybingtian@163.com (B.Y.); 2 DadHank (Chengdu) Biotech Corp., Chengdu 611130, China; yaojunli@126.com

**Keywords:** porcine-derived lactic acid bacteria, *Lactobacillus plantarum*, *Lactobacillus amylovorus*, *Lactobacillus salivarius*, biological characteristics, whole-genome sequencing, probiotics, antibiotic-free breeding

## Abstract

To screen high-quality porcine-derived lactic acid bacteria for swine production, this study compared growth performance, acid production, acid and bile salt tolerance, and genome characteristics of *Lactobacillus plantarum* (MRS002), *Lactobacillus amylovorus* (MRS003), and *Lactobacillus salivarius* (MRS004). All experiments were performed with three biological replicates, and data were analyzed using the GraphPad Prism software 8.4.3 by one-way analysis of variance (one-way ANOVA) followed by Tukey’s multiple comparison test (significance level *p* < 0.05). All three strains showed typical anaerobic growth. *L. amylovorus* MRS003 had a longer growth cycle and higher biomass, while *L. plantarum* MRS002 and *L. salivarius* MRS004 grew faster and produced more acid, with pH values reaching 4.2 and 4.3 at 24 h, respectively. *L. plantarum* MRS002 and L. salivarius MRS004 also exhibited higher survival rates under 0.3% bile salt and pH 2.0 stress. Genome annotation revealed that more than 50% of genes were related to metabolism in all strains. *L. plantarum* MRS002 possessed the most comprehensive metabolic and stress-resistance gene networks; *L. amylovorus* MRS003 shows genomic enrichment in starch-degradation pathways and appears promising for high-starch feed fermentation; and *L. salivarius* MRS004 showed unique advantages in aromatic amino acid metabolism (e.g., phenylalanine, tyrosine, and tryptophan biosynthesis). In summary, based on the evaluated in vitro indicators, *L. salivarius* MRS004 has more favorable phenotypic characteristics, *L. plantarum* MRS002 has broad adaptability, and *L. amylovorus* MRS003 is suitable for high-starch feed fermentation. This study provides a theoretical basis for the research and development of probiotic products.

## Simple Summary

With the ban on antibiotics in swine farming, there is an urgent need for safe and efficient porcine-derived probiotics. This study aimed to compare the growth, acid production, acid and bile salt tolerance, and genomic characteristics of three Lactobacillus strains: *L. plantarum*, *L. amylovorus*, and *L. salivarius*. Results showed that *L. salivarius* and *L. plantarum* had stronger acid production and gastrointestinal tolerance. Genomic analysis revealed diverse metabolic and stress resistance genes. *L. salivarius* showed the best probiotic potential, while *L. amylovorus* was suitable for starch utilization. These strains can be used to develop probiotics for pigs, improve intestinal health, reduce disease, and support antibiotic-free breeding, which benefits animal welfare and sustainable livestock production.

## 1. Introduction

Lactic Acid Bacteria (LAB) are Gram-positive bacteria that ferment carbohydrates to produce large amounts of lactic acid, with important applications in food fermentation, biological preservation, and host health regulation. As strain-specific Generally Recognized as Safe (GRAS) or Qualified Presumption of Safety (QPS) status microorganisms, many well-characterized LAB strains have been developed as dietary and feed additives, playing key roles in improving intestinal microecology and host health. With the global trend of reducing and banning antibiotics in swine production, screening, identifying, and developing porcine-derived probiotic LAB with independent intellectual property rights has become a core strategy to replace antibiotics, protect intestinal health, and promote growth performance in piglets [1].

Porcine-derived LAB isolated from the intestinal mucosa and contents of healthy pigs exhibit natural adaptation to the host’s gastrointestinal environment, with superior colonization efficiency, immune regulation, and functional stability compared with exogenous strains, making them ideal candidates for swine-specific probiotics. Studies have confirmed that porcine-derived *Lactobacillus salivarius* efficiently colonizes the ileal mucosa, promotes nutrient digestion and absorption, and regulates both innate and adaptive immune responses [2]. Genomic and metabolomic analyses further reveal that porcine *L. salivarius* harbors abundant metabolic genes encoding digestive enzymes, stress-response proteins, and cell wall stability-related factors, with no detected virulence factors, ensuring high safety. Meanwhile, tolerance to acid, bile salts and gastrointestinal stress is a prerequisite for probiotic function and a core indicator for strain screening [3].

Among porcine LAB, *Lactobacillus plantarum*, *Lactobacillus amylovorus* and *Lactobacillus salivarius* have attracted wide attention due to their unique metabolic characteristics and prominent probiotic potential. *L. plantarum* is a versatile strain widely distributed in the pig intestine and fermented feed, with broad substrate utilization and high metabolic plasticity, exhibiting antibacterial, growth-promoting and fermentation-adaptive properties [3]. *L. amylovorus* is an amylolytic LAB (ALAB) that secretes extracellular amylases to directly degrade starchy materials, showing unique advantages in high-starch fermented feed for pigs [4]. As a dominant indigenous symbiont in the porcine digestive tract, *L. salivarius* has been verified to possess excellent gastrointestinal colonization, antioxidant and broad-spectrum antibacterial activities, making it a superior strain for intestinal health regulation [1].

At present, systematic and parallel comparative studies on these three porcine LAB remain insufficient. Most studies focus on functional characterization of single strains, lacking unified experimental comparisons of growth metabolism, stress tolerance and molecular functions [5]. Several key scientific questions remain unclear: ① Differences in growth kinetics and acid production, which determine application efficiency in feed fermentation and intestinal colonization. ② Variations in gastrointestinal tolerance (acid and bile salt resistance), which are essential for survival and function in vivo [6,7]. ③ Metabolic pathway differences based on genome annotation, which reveal mechanisms of carbohydrate and amino acid metabolism differentiation and support precise application [8].

Therefore, this study used *L. plantarum*, *L. amylovorus* and *L. salivarius* isolated from healthy pig intestines to systematically evaluate growth characteristics, acid production, and tolerance to acid and bile salts. Combined with whole-genome sequencing and functional annotation, we compared carbohydrate-active enzymes, functional genes and key metabolic pathways among the three strains. This study aims to clarify the phenotypic and molecular similarities and specificities of the three strains, provide data and theoretical support for their precise application in fermented feed development, probiotic preparation and antibiotic-free swine breeding, and lay a foundation for understanding the ecological adaptation of porcine LAB.

## 2. Materials and Methods

### 2.1. Bacterial Strains

The three strains (*Lactobacillus plantarum* MRS002, *Lactobacillus amylovorus* MRS003, and *Lactobacillus salivarius* MRS004) used in this study were previously isolated and preserved by our laboratory. These strains were obtained from the colonic digesta of healthy growing Duorc × Landrace × Yorkshire (DLY) pigs. Samples were collected from eight healthy pigs under sterile conditions, and all procedures followed relevant animal care guidelines. They were provided and identified by the Institute of Animal Nutrition, Sichuan Agricultural University. All strains were stored at −80 °C in 20% (*v*/*v*) glycerol solution until use.

### 2.2. Reagents, Supplies and Instruments

Reagents: Modified MRS broth powder, L-cysteine hydrochloride, vancomycin, cefoperazone, bovine bile salt, resazurin, agar, Gram staining kit, sterile glycerol, sterile saline, etc. Supplies: Anaerobic jars, anaerobic gas packs, sterile centrifuge tubes, Petri dishes, pipette tips, sterile sampling tubes, inoculation loops, etc. Instruments: Laminar flow hood, constant-temperature anaerobic incubator, autoclave, light microscope, pH meter, UV–visible spectrophotometer, PCR amplifier, gel imaging system, etc.

### 2.3. Strain Activation, Culture and Preliminary Identification

#### 2.3.1. Medium Preparation

Modified MRS medium: A total of 55.4 g of modified MRS powder was dissolved in 1000 mL of distilled water, supplemented with 0.5 g/L L-cysteine hydrochloride and 0.0001% resazurin, and adjusted to pH 6.2–6.4. Colistin (10–20 μg/mL) was added to inhibit Gram-negative bacteria. Solid medium contained 1.5% agar and was autoclaved at 121 °C for 20 min. After cooling to 50–55 °C, plates were poured in an anaerobic workstation and incubated anaerobically for 24–48 h to confirm an oxygen-free environment.

#### 2.3.2. Sample Pretreatment

In an anaerobic workstation, 1 mL of frozen strain was mixed with 9 mL of sterile deoxygenated saline, vortexed thoroughly to prepare a 1:10 (*w*/*v*) suspension (10^−2^ dilution), and serially diluted to 10^−4^, 10^−5^ and 10^−6^.

#### 2.3.3. Inoculation and Incubation

A 0.1 mL aliquot of each dilution (10^−4^, 10^−5^, 10^−6^) was spread onto modified MRS agar plates supplemented with antibiotics, with three replicates per dilution. Plates were incubated anaerobically at 37 °C for 48–72 h.

#### 2.3.4. Screening and Purification

Putative LAB were selected based on colony morphology and Gram staining: typical colonies were circular, smooth, opaque, milky white or light yellow, 1–3 mm in diameter, with regular edges. Cells were Gram-positive, non-spore-forming rods, occurring singly, in pairs or in short chains. Isolates with consistent characteristics were purified by streak plate method and incubated anaerobically/microaerophilically at 37 °C for 24–48 h.

#### 2.3.5. Strain Preservation

Single purified colonies were inoculated into modified MRS broth and incubated anaerobically at 37 °C to logarithmic phase. Then 800 μL of culture was mixed with 200 μL of sterile glycerol (final concentration 20%), labeled, and stored at −80 °C.

#### 2.3.6. Molecular Identification by 16S rRNA Gene

After 48 h of activation, genomic DNA was extracted. The 16S rRNA gene was amplified using universal primers 27F (5′-AGAGTTTGATCMTGGCTCAG-3′) and 1492R (5′-TACGGYTACCTTGTTACGACTT-3′). The 25 μL reaction mixture: 10× PCR Buffer 2.5 μL, 2.5 mmol/L dNTPs 2 μL, 10 μmol/L forward and reverse primers 1 μL each, 5 U/μL Taq polymerase 0.2 μL, template DNA 1 μL, and ddH_2_O up to 25 μL. PCR conditions: pre-denaturation at 95 °C for 5 min; 30 cycles of 95 °C for 30 s, 55 °C for 30 s, 72 °C for 1 min; final extension at 72 °C for 10 min. PCR products were verified by 1% agarose gel electrophoresis (target fragment ~1500 bp) and sequenced by the Sanger method. Species identification was confirmed by a BLAST search in GenBank with sequence similarity ≥ 97%, and average nucleotide identity (ANI) ≥ 95% from whole-genome sequencing data against type strains.

### 2.4. Determination of Biological Characteristics

#### 2.4.1. Growth Curve

Frozen strains were streaked and activated. Single colonies were inoculated into 5 mL modified MRS broth and incubated anaerobically at 37 °C to OD_600_ ≈ 0.6–0.8 as seed culture. A 1% inoculum was transferred to 50 mL fresh medium and incubated anaerobically at 37 °C. The OD_600_ was measured at 0, 2, 4, 6, 8, 12, 24, 36 and 48 h, with three replicates per strain. Growth curves were plotted with time as abscissa and OD_600_ as ordinate. Three independent biological replicates were performed for each strain. During sampling, cultures were briefly taken and immediately returned to anaerobic conditions to avoid oxygen exposure.

#### 2.4.2. Organic Acid Synthesis Characteristics

At 0, 12, 24, 36 and 48 h, cultures were centrifuged at 12,000 r/min for 10 min. The supernatant pH was determined using a pH meter, with three replicates per time point.

#### 2.4.3. Adaptability to Exogenous Bile Salt Stress

Modified MRS broths containing 0.1%, 0.3% and 0.5% bovine bile salt were prepared, with bile salt-free medium as control. Logarithmic-phase cultures were adjusted to OD_600_ = 0.8, inoculated at 1% into each medium, and incubated anaerobically at 37 °C. Samples were taken at 0, 2, 4 and 6 h, diluted, plated and counted. Survival rate was calculated as: Survival rate (%) = (Viable count of treatment group/Viable count of control group) × 100%. Three replicates were performed per concentration.

#### 2.4.4. Survival Performance Under Low-pH Stress

Hydrochloric acid (HCl) was used to adjust the pH of sterile saline to 2.0, 3.0, and 4.0, respectively, with pH 7.0 as the control. One milliliter of logarithmic-phase culture (OD_600_ = 0.8) was mixed with 9 mL of acidic solution and incubated at 37 °C for 1 h and 2 h. Samples were diluted, plated and counted. Survival rate was calculated as above, with three replicates per pH.

### 2.5. Whole-Genome Sequencing and Functional Annotation

#### 2.5.1. Genomic DNA Extraction

Five milliliters of logarithmic-phase culture was centrifuged at 12,000 r/min for 5 min. Total DNA was extracted using a commercial bacterial genomic DNA extraction kit and stored at −20 °C.

#### 2.5.2. Library Construction and Sequencing

Genomic library construction and high-throughput sequencing were performed by a third-party company. Raw reads were filtered to remove low-quality reads, adapters and contaminants to obtain clean data. Genome assembly was performed using the de novo strategy.

#### 2.5.3. Gene Prediction and Functional Annotation

Coding sequences (CDS) were predicted using Prodigal v2.6.3. rRNA and tRNA genes were identified using RNAmmer and tRNAscan-SE, respectively. Functional annotation was performed by alignment against Swiss-Prot, TrEMBL and KEGG databases.

## 3. Results

### 3.1. 16S rRNA Identification and Phylogenetic Analysis

Following PCR amplification of the 16S rRNA gene, Sanger sequencing, and BLAST alignment against the NCBI GenBank database, all three tested strains showed ≥97% sequence homology with the corresponding type strains. Combined with morphological, physiological, and biochemical characteristics, accurate species identification was achieved:

- Strain MRS002: *Lactobacillus plantarum*;
- Strain MRS003: *Lactobacillus amylovorus*;
- Strain MRS004: *Lactobacillus salivarius*.

**Figure 1:**
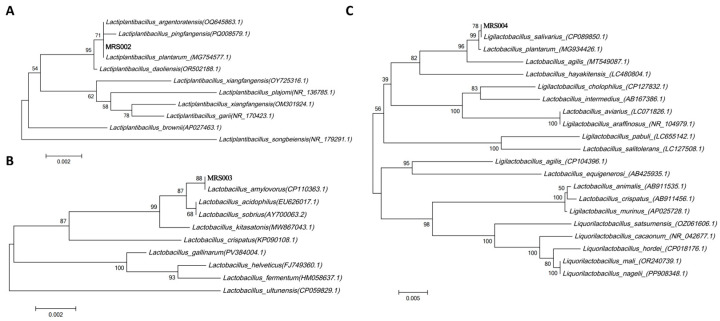
Phylogenetic tree of three tested strains based on 16S rRNA gene sequences. (**A**): Phylogenetic position of *Lactobacillus plantarum* MRS002. (**B**): Phylogenetic position of *Lactobacillus amylovorus* MRS003. (**C**): Phylogenetic position of *Lactobacillus salivarius* MRS004.

A phylogenetic tree was constructed using reference sequences of type strains. The results showed that MRS002, MRS003, and MRS004 clustered into the evolutionary branches of *L. plantarum*, *L. amylovorus*, and *L. salivarius*, respectively. The taxonomic classification and phylogenetic relationships of each strain were clear, and no cross-contamination was observed. Therefore, these strains were suitable for subsequent studies of biological characteristics and comparative genomic analysis. The phylogenetic tree is presented in Figure 1.

### 3.2. In Vitro Biological Characteristics

#### 3.2.1. Varied Proliferation Patterns of Three Porcine Lactobacilli In Vitro

**Figure 2:**
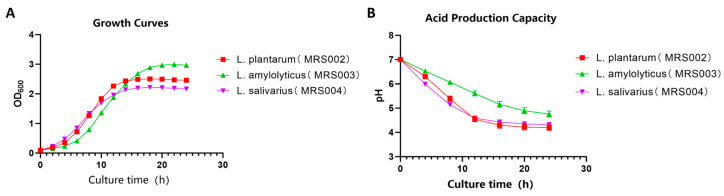
Growth curves and acid production capacity of three strains cultured in modified MRS medium. (**A**): Growth curves of *Lactobacillus plantarum* MRS002, *Lactobacillus amylovorus* MRS003, and *Lactobacillus salivarius* MRS004. (**B**): Acid production capacity (pH changes) of the three strains during anaerobic cultivation.

All three porcine lactic acid bacteria exhibited typical anaerobic growth curves in modified MRS medium, including lag, logarithmic, and stationary phases, with distinct growth kinetics. *Lactobacillus salivarius* MRS004 and *Lactobacillus plantarum* MRS002 entered the logarithmic phase rapidly at 4 h after inoculation and reached stationary phase at 15 h, showing a short lag phase and short overall growth cycle, but relatively low final biomass. *Lactobacillus amylovorus* MRS003 grew slowly with a longer lag phase, entering the logarithmic phase at 6 h and stationary phase at 20 h. Although its growth cycle was longer, it accumulated significantly higher biomass than the other two strains at 24 h (one-way ANOVA, *p* < 0.0001; Tukey’s test: *L. amylovorus* MRS003 vs. *L. plantarum* MRS002 and *L. amylovorus* MRS003 vs. *L. salivarius* MRS004, both *p* < 0.0001). The growth curves of the three strains are shown in Figure 2A.

#### 3.2.2. Acid Production Capacity

Under anaerobic cultivation, all three strains showed strong acid production and continuously decreased medium pH in a growth-dependent manner. *L. plantarum* MRS002 and *L. salivarius* MRS004 exhibited rapid and high-strength acidification, with pH dropping to 4.2 and 4.3 at 16 h, respectively, and remaining stable thereafter, which were significantly lower than that of *L. amylovorus* MRS003. One-way ANOVA revealed a significant overall difference among the three strains (*p* = 0.0002). Tukey’s multiple comparisons test further indicated that the pH values of *L. amylovorus* MRS003 were significantly higher than those of *L. plantarum* MRS002 (*p* = 0.0001) and *L. salivarius* MRS004 (*p* = 0.0004). *L. amylovorus* MRS003 produced acid more slowly and reached a stable pH of 4.8 at 20 h, indicating weaker acid production ability overall. Acid production profiles are presented in Figure 2B. In summary, all three strains possess strong organic acid accumulation capacity, providing a basis for inhibiting pathogenic bacteria in the intestine.

#### 3.2.3. Acid Tolerance of Three Lactobacillus Strains Under Simulated Gastric Acid Conditions for Swine Probiotic Screening

**Figure 3:**
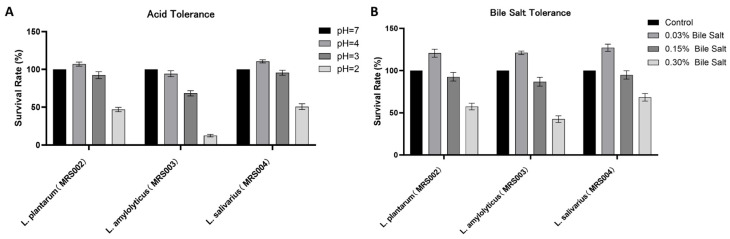
Acid tolerance and bile salt tolerance of three tested strains. (**A**): Acid tolerance of *Lactobacillus plantarum* MRS002, *Lactobacillus amylovorus* MRS003 and *Lactobacillus salivarius* MRS004 after 1 h exposure to different pH conditions. Data are presented as mean ± SD of three independent replicates. (**B**): Bile salt tolerance of *Lactobacillus plantarum* MRS002, *Lactobacillus amylovorus* MRS003 and *Lactobacillus salivarius* MRS004 after 6 h exposure to different bile salt concentrations. Data are presented as mean ± SD of three independent replicates. Significant differences were determined by one-way ANOVA followed by Tukey’s multiple comparisons test (*p* < 0.05).

In simulated gastric acid environments, all strains showed acid tolerance in varying degrees, and survival rates decreased with declining pH. Acid tolerance results are presented as mean ± SD. At pH 2.0, the survival rates were 46.8 ± 2.8% for *L. plantarum* MRS002, 12.5 ± 1.6% for *L. amylovorus* MRS003, and 50.6 ± 3.8% for *L. salivarius* MRS004. One-way ANOVA indicated a significant overall difference (*p* < 0.0001), and Tukey’s test showed that both *L. plantarum* MRS002 and *L. salivarius* MRS004 had significantly higher survival than *L. amylovorus* MRS003 (both *p* < 0.0001). At pH 3.0, survival rates were 92.3 ± 4.6%, 68.3 ± 3.5%, and 95.5 ± 3.2%, with significant differences (*p* < 0.001 **). At pH 4.0, no significant differences were detected among the three strains (** *p* > 0.05). Acid tolerance results are shown in Figure 3A. Overall, *L. plantarum* and *L. salivarius* had superior acid tolerance, better meeting the screening criteria for swine probiotics.

#### 3.2.4. Bile Salt Tolerance of Three Lactobacillus Strains for Potential Porcine Probiotic Application

Survival rates of all three strains decreased with increasing bile salt concentration. *L. plantarum* MRS002 and *L. salivarius* MRS004 maintained high survival rates in 0.1–0.3% bile salt, demonstrating excellent bile salt tolerance. *L. amylovorus* MRS003 was sensitive to bile salt stress, with survival decreasing sharply at concentrations ≥ 0.3%. Bile salt tolerance is presented in Figure 3B. These results indicate that *L. plantarum* and *L. salivarius* can better overcome the intestinal bile salt barrier and stably colonize the porcine gut to exert probiotic functions.

### 3.3. Whole-Genome Functional Annotation Analysis

#### 3.3.1. General Genome Function

**Figure 4:**
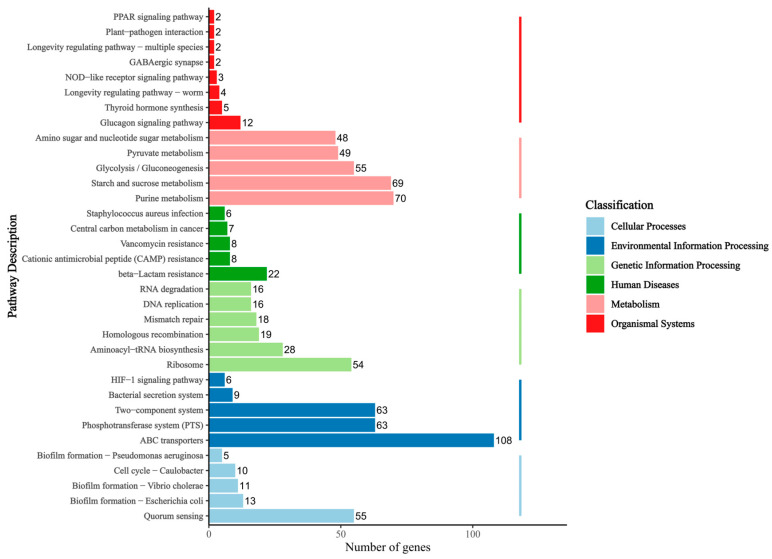
Metabolic pathway map of functional annotation for *Lactobacillus plantarum* MRS002.

**Figure 5:**
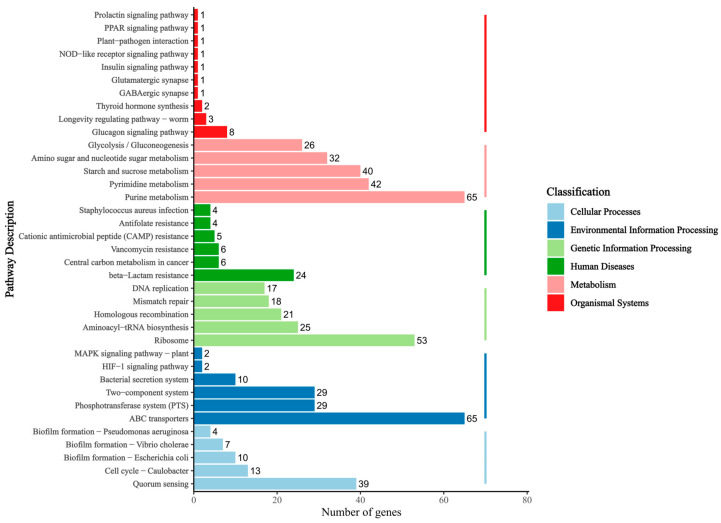
Metabolic pathway map of functional annotation for *Lactobacillus amylovorus* MRS003.

**Figure 6:**
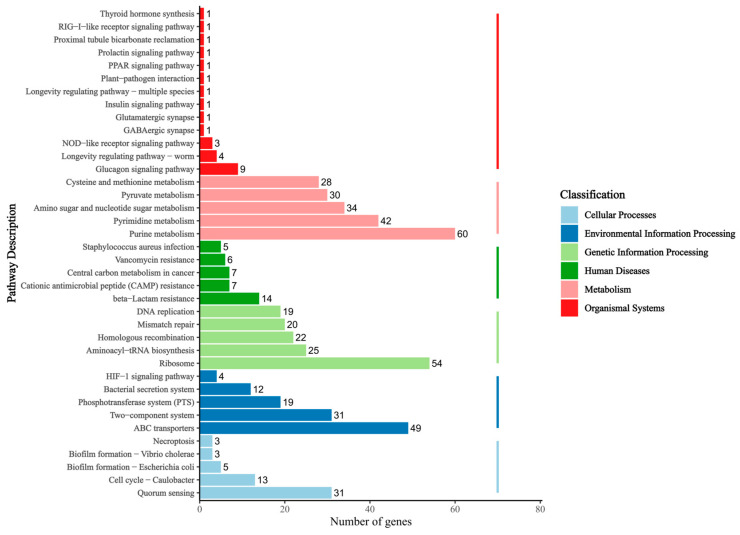
Metabolic pathway map of functional annotation for *Lactobacillus salivarius* MRS004.

KEGG pathway annotation revealed that more than 50% of genes were associated with metabolism in all three strains, indicating active metabolic functions and typical genetic characteristics of probiotic lactic acid bacteria. The overall metabolic pathway maps based on KEGG functional annotation are presented in Figure 4, Figure 5 and Figure 6 for *Lactobacillus plantarum*, *Lactobacillus amylovorus*, and *Lactobacillus salivarius*, respectively.

- *Lactobacillus plantarum* MRS002: 1669 total coding genes, 868 metabolism-related genes (52%);
- *Lactobacillus amylovorus* MRS003: 1132 total coding genes, 618 metabolism-related genes (54%);
- *Lactobacillus salivarius* MRS004: 1133 total coding genes, 569 metabolism-related genes (50%).

#### 3.3.2. Comparison of Amino Acid Metabolism Pathways

Total genes involved in amino acid metabolism were ranked as: *L. plantarum* (125) > *L. salivarius* (98) > *L. amylovorus* (73), showing distinct metabolic divergence. All three strains harbored similar numbers of aminoacyl-tRNA biosynthesis genes (19–21), supporting basal protein synthesis. The gene numbers involved in amino acid metabolism pathways of the three strains are listed in Table 1.

*L. plantarum* possessed abundant genes for alanine, aspartate, glutamate, cysteine, and methionine metabolism, with the most comprehensive network.

*L. salivarius* possessed 13 genes in the combined pathway for phenylalanine, tyrosine, and tryptophan biosynthesis, significantly more than the other two strains, indicating unique genetic potential in aromatic amino acid metabolism and host immune interaction.

*L. amylovorus* had a relatively compact amino acid metabolism network and relied more on exogenous amino acids.

#### 3.3.3. Comparison of Carbon Metabolism Pathways

Total genes involved in carbon metabolism were ranked as: *L. plantarum* (115) > *L. amylovorus* (83) > *L. salivarius* (72), reflecting different substrate preferences. Core carbon metabolism modules, including glycolysis/gluconeogenesis, TCA cycle, pyruvate metabolism, and the pentose phosphate pathway, mostly existed as combined pathways, indicating highly integrated metabolism. Differences in carbon metabolism among the three strains are presented in Table 2.

*L. plantarum* had the largest carbon metabolism gene set, with wide substrate utilization and high metabolic plasticity.

*L. amylovorus* was significantly enriched in starch and sucrose metabolism pathways, including genes encoding alpha-amylase, maltose-specific transporter, and glycoside hydrolase (GH13, GH31) family modules, supporting efficient starch breakdown.

*L. salivarius* exhibited a streamlined and efficient carbon metabolism network, well-adapted to symbiotic life in the porcine intestine.

#### 3.3.4. Colonization and Stress Resistance Pathways

Total genes related to colonization and stress resistance were ranked as: *L. plantarum* (230) > *L. amylovorus* (180) > *L. salivarius* (125), revealing differences in environmental adaptability. ABC transporters were highly enriched in all three strains (38–48), supporting nutrient uptake and metabolic homeostasis. Phosphotransferase system (PTS) gene numbers were: *L. plantarum* (41) > *L. amylovorus* (27) > *L. salivarius* (10), consistent with carbohydrate transport capacity. Data regarding colonization and stress resistance-related genes of the three strains are displayed in Table 3.

Genes for quorum sensing, biofilm formation, and drug resistance were widely distributed; *L. plantarum* had the most, indicating stronger environmental adaptation and intestinal colonization ability.

All three strains were found to harbor genes associated with beta-lactam and vancomycin resistance. Although these genes may help strains survive under harsh intestinal conditions, the presence of such antibiotic resistance determinants raises potential biosafety risks. Further investigations are required to assess their horizontal gene transfer potential and evaluate relevant safety risks prior to practical application as feed probiotics.

## 4. Discussion

Growth characteristics represent an essential foundation for the probiotic functions of lactic acid bacteria. In the present study, all three porcine-derived lactobacilli exhibited typical anaerobic growth patterns, with obvious differentiation in lag-phase duration, initiation of logarithmic phase, and biomass accumulation. These differences were closely related to their inherent metabolic traits and substrate utilization efficiency [9]. Acid production is a key functional indicator for probiotics to inhibit intestinal pathogens and maintain microecological balance. Our results demonstrated that all three strains significantly decreased medium pH via organic acid production. Notably, *L. plantarum* and *L. salivarius* showed faster acidification rates and lower final pH values, which help suppress opportunistic pathogens such as *Escherichia coli* and Salmonella and maintain intestinal health homeostasis in pigs [10].

### 4.1. Growth Characteristics

Modified MRS medium is a standard culture system for LAB and has been widely verified to efficiently support the growth of *L. plantarum*, *L. amylovorus*, and *L. salivarius* [11]. The growth kinetic profiles of the three strains in this study were consistent with previous reports. The growth and acid production of *L. plantarum* were highly coupled, and its metabolic rate was significantly affected by environmental pH, which agrees with our findings [12,13]. The longer lag phase but higher final biomass of *L. amylovorus* can be attributed to its amylolytic metabolic characteristics and energy conversion strategy.

This study only evaluated the basic biological characteristics in vitro. Future research should further verify intestinal adhesion, enzymatic production, short-chain fatty acid profiles, and conduct in vivo trials to reveal effects on intestinal barrier, immunity, and growth performance, thus providing more comprehensive data for developing high-quality porcine LAB preparations [14].

### 4.2. Acid Production Capacity

The acid production patterns observed in this study were consistent with previous reports. *Lactobacillus plantarum* presented prominent acid-producing capacity, which may be associated with its complete carbohydrate metabolic pathways. Higher lactic acid accumulation is considered capable of suppressing the growth of intestinal pathogens [15]. As native porcine intestinal commensal bacteria, *Lactobacillus salivarius* also displayed high acidification capacity, which is in line with its adaptive characteristics to the intestinal microenvironment [16,17]. In comparison, *Lactobacillus amylovorus* showed weaker acid production performance, which makes it more applicable to fermentation systems dominated by starchy raw materials [18,19,20,21,22]. Such phenotypic distinctions lay a theoretical basis for differentiated application of the three strains in feed fermentation and intestinal microecology regulation.

The acid tolerance assay used simplified acidic saline rather than simulated gastric fluid containing pepsin and electrolytes, which may reduce physiological relevance; future studies should use standardized simulated gastric fluid.

### 4.3. Bile Salt Tolerance

Bile salt tolerance is a core prerequisite for probiotics to transit the porcine small intestine and achieve intestinal colonization. Our results showed that *Lactobacillus plantarum* MRS002 and *Lactobacillus salivarius* MRS004 maintained remarkably higher survival rates under bile salt concentrations ranging from 0.1% to 0.3%, exhibiting strong bile resistance, which was consistent with previous research findings on porcine-derived lactobacilli [23,24,25,26,27,28]. By contrast, *Lactobacillus amylovorus* was more susceptible to bile salt stress, and this phenotypic difference may be linked to bacterial membrane structure as well as the carriage and transcription level of bile salt tolerance-associated genes.

Combined with our whole-genome annotation data, we confirmed the presence of classic bile salt hydrolase coding gene bsh in the two high-tolerance strains. In addition, multiple functional genes participating in stress adaptation were also identified, including peptidoglycan synthesis-related genes, phosphotransferase system (PTS) family genes, and molecular chaperone genes such as groEL and groES. These identified genetic elements can well account for the inter-strain differences in bile salt tolerance observed in this experiment, and offer reliable genetic evidence for subsequent directional strain modification and functional optimization.

### 4.4. Acid Tolerance

All three strains showed certain acid tolerance in simulated gastric fluid (pH 2.0–4.0). *L. plantarum* and *L. salivarius* exhibited higher survival under strong acidic conditions, enabling better passage through the gastric barrier. The acid tolerance mechanism of *L. plantarum* mainly relies on membrane H^+^-ATPase to expel intracellular protons and maintain pH homeostasis, while membrane fatty acid composition enhances stability under acid stress [29,30,31]. *L. amylovorus* survived well under weak acidic conditions, meeting basic intestinal survival requirements. These results confirm that all three strains possess the necessary stress resistance for swine probiotics.

### 4.5. Overall Analysis of Genome Functional Annotation

KEGG annotation revealed that all three porcine lactobacilli possessed active metabolic functions, with more than 50% of genes related to metabolism, consistent with high-quality probiotic LAB. *L. plantarum* MRS002 had the largest genome, the most genes for amino acid metabolism, carbon metabolism, colonization, and stress resistance, and the most comprehensive metabolic network, conferring stronger adaptability and regulatory potential in the host intestine. *L. amylovorus* MRS003 was enriched in starch-degradation pathways, showing outstanding substrate specificity and unique advantages in high-starch feed utilization [32,33,34,35]. *L. salivarius* MRS004 had a streamlined metabolic profile, focusing on host–microbe interaction, with prominent potential in immune regulation and intestinal homeostasis.

### 4.6. Amino Acid Metabolism

Amino acid metabolism is essential for bacterial proliferation and host nutritional interaction. The three strains harbored similar numbers of aminoacyl-tRNA biosynthesis genes, ensuring basal protein synthesis. *L. plantarum* possessed the highest total amino acid metabolism genes, with abundant distribution in pathways for alanine, aspartate, glutamate, cysteine, and methionine metabolism, indicating comprehensive synthetic capacity. Although *L. salivarius* had a moderate total number of genes, it was significantly enriched in the combined pathway for phenylalanine, tyrosine, and tryptophan biosynthesis, suggesting its ability to modulate host immunity via aromatic amino acid metabolism, a key molecular feature of an intestinal symbiont [36]. *L. amylovorus* had a relatively compact amino acid metabolism network and relied more on exogenous amino acids.

### 4.7. Carbon Metabolism

Core carbon metabolism genes mainly existed as combined pathways in the three strains, indicating high integration of glycolysis, pyruvate metabolism, the pentose phosphate pathway, and other key processes. *L. plantarum* had the most carbon metabolism genes, a wide substrate spectrum, and high metabolic plasticity, adapting to complex feed matrices. *L. amylovorus* was enriched in starch-utilization pathways, directly decomposing starchy materials and improving feed energy efficiency. *L. salivarius* had a streamlined and efficient carbon metabolism network, well-adapted to the oligosaccharide- and monosaccharide-rich environment of the porcine intestine, consistent with the energy strategy of symbiotic microorganisms.

### 4.8. Colonization and Stress Resistance Analysis

All three lactobacilli were non-motile and depended on adhesion and biofilm formation for intestinal colonization rather than flagellar movement. ABC transporters were highly enriched in all strains, supporting nutrient uptake, metabolite efflux, and stress adaptation [37]. The number of phosphotransferase system (PTS) genes followed the order *L. plantarum* > *L. amylovorus* > *L. salivarius*, consistent with their carbohydrate transport capacity. Genes related to quorum sensing and biofilm formation were most abundant in *L. plantarum*, suggesting enhanced colonization stability via group behavior. In addition, genes associated with β-lactam and vancomycin resistance were annotated in the three strains. The presence of these antibiotic resistance determinants requires further safety evaluation, including horizontal gene transfer risk assessment, prior to their application as feed-grade probiotics.

## 5. Conclusions

In this study, systematic in vitro biological characterization and whole-genome functional annotation were performed on *Lactobacillus plantarum*, *Lactobacillus amylovorus* and *Lactobacillus salivarius*. The probiotic potential and genomic functional profiles of the three strains were clarified, providing theoretical references and experimental evidence for their subsequent development as probiotic preparations and application in intestinal microecological regulation.

In vitro assays showed that all three lactobacilli displayed typical anaerobic growth patterns in MRS medium with distinct growth differences. Among them, *L. salivarius* exhibited the fastest growth rate, consistent with previous reports. All strains possessed strong acid-producing capacity and significantly reduced medium pH, which is in line with typical metabolic characteristics of lactobacilli. Organic acids secreted by the strains could help inhibit intestinal pathogenic bacteria and maintain intestinal microflora homeostasis. Regarding stress resistance, the three strains maintained viable survival under 0.1–0.5% bile salt and pH 2.0–4.0 acidic conditions, showing basic tolerance to the simplified simulated gastrointestinal stresses in this study. Several strains exhibited relatively strong acid and bile salt resistance in vitro, laying a preliminary foundation for potential intestinal colonization and probiotic function exertion.

Whole-genome sequencing and functional annotation revealed abundant functional genes in all strains, with divergent functional priorities. *L. plantarum* harbored abundant metabolism-associated genes, *L. amylovorus* held inherent advantages in starch substrate utilization, while *L. salivarius* was enriched in genes potentially related to host–microbe crosstalk. Amino acid metabolism analysis indicated similar quantities of amino acid biosynthesis genes among strains, along with conserved basic pathways including alanine, aspartate and glutamate metabolism. Notably, *L. salivarius* contained markedly more genes involved in phenylalanine, tyrosine and tryptophan biosynthesis, suggesting a preliminary genetic basis for potential host regulatory effects that require further experimental validation using metabolomic, immunological, or host-cell models.

Carbon metabolism analysis demonstrated that core pathways including glycolysis, the TCA cycle, pyruvate metabolism and the pentose phosphate pathway were highly integrated. Carbohydrate fermentation metabolism of the three strains conformed to typical metabolic traits of lactobacilli. Colonization and stress-related gene analysis showed that all strains were non-motile, lacking genes for flagellar assembly and bacterial chemotaxis. Intestinal colonization mainly depended on adhesion and non-motile strategies. Abundant ABC transporter genes supported nutrient uptake, metabolite excretion and drug resistance. Phosphotransferase system (PTS) genes followed the order *L. plantarum* > *L. amylovorus* > *L. salivarius*, matching their carbohydrate fermentation capacities. Quorum sensing and biofilm-related genes were widely distributed, especially in *L. plantarum*, enhancing intestinal colonization stability via interbacterial cooperation. In addition, all strains carried resistance genes against β-lactams and vancomycin, ensuring survival in antibiotic-containing intestinal habitats.

In conclusion, *L. plantarum*, *L. amylovorus* and *L. salivarius* all show preliminary in vitro probiotic-related traits, with strain-specific differences in growth performance, metabolic functions and stress tolerance. Their genomic features provide valuable information for understanding strain-specific characteristics and application potential. Further safety evaluation, adhesion assays, pathogen inhibition tests, and in vivo validation are still needed to fully confirm their probiotic effects and support the rational application of these strains in swine production.

## Figures and Tables

**Table 1:** Amino acid metabolism analysis of the three strains.

Pathway Name	*L. amylovorus*	*L. plantarum*	*L. salivarius*
Aminoacyl-tRNA biosynthesis	19	21	19
Aminoacyl-tRNA biosynthesis; ...	4	4	4
Alanine, aspartate and glutamate metabolism	1	1	1
Alanine, aspartate and glutamate metabolism; ...	7	12	7
Arginine biosynthesis	0	0	0
Arginine biosynthesis; ...	4	9	5
Arginine and proline metabolism	2	2	1
Arginine and proline metabolism; ...	1	4	1
Cysteine and methionine metabolism	1	1	1
Cysteine and methionine metabolism; ...	9	18	13
Glycine, serine and threonine metabolism	0	1	0
Glycine, serine and threonine metabolism; ...	12	18	11
Histidine metabolism	0	0	0
Histidine metabolism; ...	0	11	0
Lysine biosynthesis	0	0	0
Lysine biosynthesis; ...	11	12	10
Lysine degradation	0	0	0
Lysine degradation; ...	1	2	3
Phenylalanine, tyrosine and tryptophan biosynthesis	0	0	0
Phenylalanine, tyrosine and tryptophan biosynthesis; ...	1	7	13
Valine, leucine and isoleucine biosynthesis	0	0	0
Valine, leucine and isoleucine biosynthesis; ...	1	1	1
Total (Deduplicated Sum)	73	125	98

**Table 2:** Carbon metabolism analysis of the three strains.

Pathway Name	*L. amylovorus*	*L. plantarum*	*L. salivarius*
Glycolysis/Gluconeogenesis	0	0	0
Glycolysis/Gluconeogenesis; ...	17	33	18
Citrate cycle (TCA cycle)	0	0	0
Citrate cycle (TCA cycle); ...	6	7	4
Pyruvate metabolism	1	2	1
Pyruvate metabolism; ...	8	18	10
Pentose phosphate pathway	1	2	1
Pentose phosphate pathway; ...	9	17	9
Carbon metabolism	0	0	0
Carbon metabolism; ...	1	1	1
Glyoxylate and dicarboxylate metabolism	0	1	0
Glyoxylate and dicarboxylate metabolism; ...	4	7	5
Propanoate metabolism	0	0	0
Propanoate metabolism; ...	3	5	4
Butanoate metabolism	0	0	0
Butanoate metabolism; ...	2	3	2
Methane metabolism	0	0	0
Methane metabolism; ...	7	10	8
Other carbon fixation pathways	0	0	0
Other carbon fixation pathways; ...	7	10	5
Total (Deduplicated Sum)	83	115	72

**Table 3:** Colonization and stress resistance analysis of the three strains.

Pathway Name	*L. amylovorus*	*L. plantarum*	*L. salivarius*
Bacterial chemotaxis	0	0	0
Bacterial chemotaxis; ...	0	0	1
Flagellar assembly	0	0	0
Flagellar assembly; ...	0	0	0
Quorum sensing	9	5	2
Quorum sensing; ...	16	30	18
Biofilm formation—*Pseudomonas aeruginosa*	0	0	0
Biofilm formation—*Vibrio cholerae*	1	1	2
Biofilm formation—*Escherichia coli*	1	2	0
Biofilm formation—...; ...	7	8	3
beta-Lactam resistance	2	2	1
beta-Lactam resistance; ...	17	16	9
Vancomycin resistance	0	0	0
Vancomycin resistance; ...	4	8	4
Cationic antimicrobial peptide (CAMP) resistance	0	1	1
Cationic antimicrobial peptide (CAMP) resistance; ...	5	7	6
ABC transporters	48	45	38
ABC transporters; ...	17	10	3
Phosphotransferase system (PTS)	6	8	2
Phosphotransferase system (PTS); ...	21	33	8
Two-component system	6	22	7
Two-component system; ...	14	19	9
Oxidative phosphorylation	1	3	2
Oxidative phosphorylation; ...	9	11	10
Total (Deduplicated Sum)	180	230	125

## Data Availability

The original contributions presented in this study are included in the article. Further inquiries can be directed to the corresponding author.
